# Using Full Pose Measurement for Serial Robot Calibration

**DOI:** 10.3390/app12073680

**Published:** 2022

**Authors:** Marek Franaszek, Jeremy A. Marvel

**Affiliations:** Intelligent Systems Division, National Institute of Standards and Technology, Gaithersburg, MD 20899, USA

**Keywords:** robot calibration, robot remastering, calibration uncertainty, part probing, uncertainty reduction, sensor feedback

## Abstract

To ensure smooth robot operations, parameters of its kinematic model and a registration transformation between robot base and world coordinate frame must be determined. Both tasks require data acquired by external sensors that can measure either 3D locations or full 6D poses. We show that use of full pose measurements leads to much smaller robot orientation errors when compared with the outcome of calibration and registration procedures based on 3D data only. Robot position errors are comparable for both types of data. The conclusion is based on extensive simulations of 7 degrees of freedom robot arm and different levels of pseudo-noise perturbing both positional and rotational components of pose.

## 1. Introduction

The topic of robot calibration is well-established, yet it is still a significant factor identified by end-users as being negatively impactful for robot usability and utility [1]. Calibration is followed by registration of robot frame to world frame so the accurate encoder angles can be obtained from inverse kinematic and fed to the robot’s controller. Both procedures have a profound impact on robot performance and, as pointed out in [2], “it is impossible to distinguish the end-effector error contributed either by” incorrect model parameters or by inaccurate registration transformation.

Various methods of calibrating a robot’s kinematic chain have been developed (e.g., [3-5]). Many of these methods rely on intrinsic kinematic models (e.g., [6-9]), which minimize complicated, nonlinear error functions (unless only linearized error models are considered, which may exchange uncertainty for mathematical simplicity) in at least *N*-dimensional space, where *N* is the number of controllable joints in the serial kinematic chain. Calibrations based on extended modeling (i.e., beyond rigid kinematics) include compensating for thermal effects [10], and elastostatic [11] and higher order errors [12]. Likewise, examples of non-kinematics-based calibrations can be seen in [13,14]. There are also compensation techniques that can handle both kinematic and non-kinematic errors, but they require steady calculations and application of corrections during on-line operations [15-17], or dynamically selected pre-calculated, hand–eye calibrations from a table [18].

Robot calibration procedures depend on theoretical models of the mechanical system’s forward kinematic. For a serial open chain robot, the Product of Exponentials (POE)—based on screw theory—is thought to be one of the most versatile models that can handle singularities in the popular Denavit–Hartenberg (DH) parameter model [19]. For robots with revolute joints only, each joint is parametrized by a three-dimensional (3D) unit vector indicating axis of rotation, and a 3D vector of any point on the axis line. Calibration procedures for such models rely on Circle Point Analysis (CPA) applied to 3D data acquired with laser tracker or other sensor: positioning the robot into a zero-reference configuration (i.e., where all joint angles are set to zero), and then rotating each joint one by one while keeping all other joints fixed at zero [20,21]. Unfortunately, POE-based models do not explicitly include zero offsets of encoder angles. Accurate estimating of zero offsets is critical because the largest contribution to the robot positioning error (97%) comes from incorrect zero offsets [22]. Performing the zero offsets calibration in CPA causes that errors in registration transformation and in the individual offsets accumulate. This may lead to inconsistent calibration results. For some poses, the calibration process reduced robot pose error seven-fold; for others, it actually increased error twofold [23].

A desired outcome of calibration is the error reduction in full pose of robot end-effector, i.e., in its position and orientation. However, both components belong to two different spaces: position is a vector in 3D space and its components have length units, like millimeters, while orientation matrix is parameterized by three angles in degrees. This causes a fundamental scaling problem when a full pose error is minimized (as discussed in [24], ad hoc introduced scaling factors put more weight either on linear or angular part of pose error and push optimizer towards different solutions). This may become a problem in commercial applications where not only position but also orientation of end-effector is important. For example, in automated drilling, a parallelism between the spindle axis and the normal axis of the drilling plate surface should be below 0.2° [25]. Small orientation error 0.05° required for automated riveting, drilling and spot welding was demonstrated by applying online pose corrections in [26]. Automated fiber placement is another example of industrial application where the orientation of robot end-effector is important [27].

The approach that we introduce in this paper avoids the pitfall of minimization of unbalanced 6D error. First, link twists are determined in the CPA-like procedure from 3D data. Then, using full 6D poses measured by sensors, encoder zero offsets are determined in a separate minimization. The error function used in this minimization does not depend on linear DH parameters (link lengths and linear offsets) nor on the position components of noisy 6D poses acquired by sensors. Once twists and zero offsets are known, they are inserted into another error function, which depends only on position components of sensor data. The remaining linear DH parameters are determined by minimizing this second error function. For comparison, robot calibration based on only 3D sensor data is also performed. Obtained results clearly show that orientation errors of end-effector are smaller when orientation part of 6D data is used. At the same time, the position errors are comparable for both methods.

## 2. Background

The frame ***F***_*TCP*_ associated with the robot’s Tool Center Point (TCP) coordinate system can be expressed as a 4 × 4 homogeneous transformation consisting of a 3 × 3 rotation matrix ***R*** and a 3 × 1 translation vector ***t***:

(1) $$F_{TCP}=\left[\begin{array}{cc} R_{3\times 3} & t_{3\times 1}\\ 0_{1\times 3} & 1 \end{array}\right]$$

For a serial, open-chain collaborative robot arm with *N* revolute joints, the frame ***F**_TCP_* in the robot’s base coordinate system can be determined using a forward kinematic model:

(2) $$F_{TCP}(\theta_{k})=F_{1}F_{2}\ldots F_{N}F_{T}$$

where

(3) $$\theta_{k}=[\theta_{1,k},\theta_{2,k},\ldots ,\theta_{n,k},\ldots ,\theta_{N,k}]$$

and *θ_n,k_* is the encoder angle of the *n*-th revolute joint for the *k*-th robot configuration, ***F**_n_* is the homogeneous transformation associated with the *n*-th joint, *n* = 1, …, *N*, and ***F**_T_* is a transformation from the robot’s flange frame to the TCP. Using the nominal DH parameters, the rotation component of each ***F**_n_* can be written as

(4) $$R_{n,k}(\theta_{n,k},\epsilon_{n},\alpha_{n})=R_{z}(\theta_{n,k}+\epsilon_{n})R_{x}(\alpha_{n})$$

where ***R**_z_* and ***R**_x_* are rotations around *z* and *x* axis, respectively. Two angular DH parameters in (4) are *α_n_* (the link twist) and *ϵ_n_* (the zero offset angle for the *n*-th encoder). The translation component of ***F**_n_* can be expressed as

(5) $$t_{n}={\left[\;\;r_{n}\;\cos(\theta_{n,k}+\epsilon_{n})\;\;r_{n}\;\sin(\theta_{n,k}+\epsilon_{n})\;\;d_{n}\;\;\right]}^{T},$$

where *r_n_* and *d_n_* are two linear DH parameters (link length and offset), and […]^*T*^ denotes the vector transpose.

From (2) and (4) it can be seen that the rotation part ***R***_*TCP*_ of the ***F***_*TCP*_ frame depends only on the rotation components

(6) $$R_{TCP}(\theta_{k})=R_{1,k}R_{2,k}\ldots R_{N,k}R_{T}.$$

This is a general property of serial chain manipulators with revolute joints, and is not dependent on a particular kinematic model (here, we use the DH model for illustration purposes only). In the remainder of this paper, we use the notation

(7) $$R_{k}=R_{TCP}(\theta_{k},\alpha ,\epsilon )$$

where ***α*** = [*α*_1_, *α*_2_, …, *α_N_*] and *ϵ* = [*ϵ*_1_, *ϵ*_2_, …, *ϵ_N_*] are the vectors of the DH angular parameters. Note that the positional component ***t***_*k*_ of the ***F***_*TCP*_ (***θ***_*k*_) frame depends on joint angles and all four vectors of the DH parameters:

(8) $$t_{k}=t_{TCP}(\theta_{k},\alpha ,\epsilon ,r,d).$$

## 3. Determination of Link Twist

To ensure that forward kinematics correctly predict the tool pose in the robot coordinate frame, the DH parameters must be determined first during the robot calibration process. Once calibrated, they remain fixed during robot on-line operations. Calibration may be performed by installing a spherically-mounted retro-reflector (SMR) at the robot’s TCP, and tracking it with a laser tracker. From four vectors of DH parameters (***α, ϵ, r, d***), the twist angles ***α*** can be determined independently of other DH parameters by using 3D data acquired for CPA procedure. The twist angle *α_n_* is defined as the angle between two consecutive joint axes of rotation, ***u**_n_* and ***u***_*n*+1_ (the last twist *α_N_* is, by definition, set to zero). If ***C***_*n,K*_ denotes a set of *K* 3D points ***t***_*k*_ calculated in (8) and acquired for *n*-th joint in CPA procedure, then these points are distributed along an arch (section of a circle) on a plane in 3D space. Thus, for each joint *n* = 1, …, *N*, a unit vector ***c**_n_* normal to the fitted plane can be calculated. While the exact locations of ***C***_*n,K*_ points depend on all DH parameters (***ϵ, α, r, d***), vector ***c***_*n*_ is parallel to the axis of rotation ***u**_n_* and, therefore, *α_n_* can be determined from the scalar product of two consecutive axes, *α_n_* = arccos (***c**_n_* · ***c***_*n*+1_). If ***B***_*n,K*_ is the set of 3D points measured by laser tracker which correspond to ***C***_*n,K*_, then a unit vector ***b***_*n*_ normal to the plane fitted to ***B**_n,K_* can be calculated and the angle between two consecutive ***b****_n_* and ***b***_*n*+1_ is used as the estimate of *α_n_*.

To get correctly estimated twist angles *α_n_*, two important steps must be followed. First, since arccos () is an even function, a sign of estimated angle must be equal to the sign of the default (i.e., theoretical) twist angle, *sign*(*α*_0,*n*_). Second, plane fitting procedure provides only a normal to the plane, its particular direction (up or down) depends on a bounding box containing the points. To remove this ambiguity, fitted normal ${\tilde{b}}_{n}$ must obey the right hand rule together with acquired 3D points ***B***_*n,K*_, which are located on a section of a circle. Thus, the estimated corrected twist angle ${\tilde{\alpha }}_{n}$ is determined as

(9) $${\tilde{\alpha }}_{n}=sign(\alpha_{0,n})\;\text{arccos}\;({\tilde{b}}_{n}\cdot {\tilde{b}}_{n+1}).$$

## 4. Robot Calibration Based on 3D Measurements

Once the twist angles $\tilde{\alpha }$ are estimated, they can be inserted in (4) and the remaining DH parameters (***ϵ, r, d***) can be found in traditional calibration procedure using 3D data. Given *K* configurations of the arm (i.e., “poses”) defined by ***θ**_k_* = (*k* = 1, …, *K*), the SMR is moved to *K* positions ***g**_k_* = [*x_k_*, *y_k_*, *z_k_*] in 3D Cartesian space. Since ***g**_k_* and ***t**_k_* in (8) are determined in different coordinate frames, the error function *Err_pos_* in the calibration process is based on relative distances between two 3D points to avoid a dependence on a registration. For convenience, the whole set of *K* points can be divided in two halves and then

(10) $$Err_{pos}(\tilde{\alpha },\epsilon ,r,d)=\frac{1}{K∕2}\sum_{k=1}^{K∕2}(L_{k}(\tilde{\alpha },\epsilon ,r,d)-h_{k}{)}^{2}$$

where

(11) $$L_{k}(\tilde{\alpha },\epsilon ,r,d)=\|t_{k}-t_{k+K∕2}\|$$

and ***t**_k_*, ***t***_*k+K*/2_ are determined in (8), ∣∣… ∣∣ is the Euclidean norm and *h_k_* = ∣∣***g**_k_* − ***g***_*k*+*K*/2_∣∣ is a distance between two points measured by laser tracker.

Thus, the fitted DH parameters ($\tilde{\epsilon }$, $\tilde{r}$, $\tilde{d}$) can be estimated by minimizing *Err_pos_*

(12) $$(\tilde{\epsilon },\tilde{r},\tilde{d})=\underset{\epsilon ,r,d}{\text{min}}Err_{pos}(\tilde{\alpha },\epsilon ,r,d).$$

providing the vector of link twist angles $\tilde{\alpha }$ is known. The actual dimension of search space is 3*N* − 2 since the distance between two points in (11) does not depend on *d*_1_ and *ϵ*_1_ (the two parameters may have arbitrary values which only affect the registration transformation between robot and sensor). In the remainder of this paper, we call this procedure Method 1.

## 5. Calibration Based on 6D Measurements

Such data were used for robot calibration using different procedures [14,28,29]. The approach we propose calculates zero offsets $\tilde{\epsilon }$ in a separate minimization based on orientation components of 6D poses and determined earlier twist angles $\tilde{\alpha }$.

In the remainder of this paper, we assume that, for each robot configuration defined by ***θ***_*k*_, there is a corresponding 3 × 3 rotation matrix ***G**_k_* provided by an external sensor. Both ***G**_k_* and ***R**_k_* in (7) are determined in different coordinate frames. If **Ω** denotes the rotation component of registration matrix then, for each *k*-th robot orientation ***R**_k_* in (7) and the corresponding ***G**_k_* measured with the external sensor, the following relation holds:

(13) $$G_{k}=N_{k}\;\Omega \;R_{k}$$

where $N_{k}$ is a small, random rotation accounting for noise in the orientation part of 6D data acquired by sensors. For a pair of orientations ***G**_k_* and *G_k′_* (where *k′* = *k* + *K*/2), matrix ***D**_k_* can be defined as

(14) $$D_{k}=G_{k}R_{k}^{-1}R_{k^{\prime }}G_{k^{\prime }}^{-1}=N_{k}N_{k^{\prime }}^{-1}$$

and its angle of rotation *ψ_k_* ∈ [0° 180° ] is calculated as

(15) $$\psi_{k}=\text{arccos}\left(\frac{1}{2}(trace(D_{k})-1)\right).$$

Matrix ***D**_k_* and its angle *ψ_k_* depend on the measured joint angle vectors ***θ***_*k*_ and ***θ****_k′_*, the twist angles $\tilde{\alpha }$ estimated earlier, and all zero offsets *ϵ_n_* for *n* = 2, …, *N*, which can be obtained by minimizing the error function *Err_rot_*

(16) $$({\tilde{\epsilon }}_{2},\ldots ,{\tilde{\epsilon }}_{N})=\underset{\epsilon_{2},\ldots ,\epsilon_{N}}{\text{min}}Err_{rot}(\tilde{\alpha },\epsilon_{2},\ldots ,\epsilon_{N}),$$

where

(17) $$Err_{rot}(\tilde{\alpha },\epsilon_{2},\ldots ,\epsilon_{N})=\frac{1}{K∕2}\sum_{k=1}^{K∕2}\psi_{k}(\tilde{\alpha },\epsilon_{2},\ldots ,\epsilon_{N}).$$

Once the zero offsets $\tilde{\epsilon }$ are estimated, they can be inserted in (12) and the linear DH parameters ***r*** and (*d*_2_, …, *d_N_*) can be found by minimizing *Err_pos_*($\tilde{\alpha }$, $\tilde{\epsilon }$, ***r, d***) in (10). In the remainder of this paper, we call this procedure Method 2.

To show a scaling problem when both position and rotation errors are simultaneously minimized, robot calibration was attempted by minimizing the following error function:

(18) $$Err_{full}(\tilde{\alpha },\epsilon ,r,d)=Err_{pos}+wErr_{rot},$$

where *Err_pos_* is defined in (10), *Err_rot_* in (17) and positive scaling factor *w* ensures correct dimensionality of *Err_full_*. In the remainder of this paper, we call this procedure Method 3.

## 6. Registering Robot Frame

When all robot model parameters are known, i.e., estimated ${\tilde{\epsilon }}_{2}$, …, ${\tilde{\epsilon }}_{N}$, $\tilde{\alpha }$, $\tilde{r}$, ${\tilde{d}}_{2}$, …, ${\tilde{d}}_{N}$ and arbitrary values are assigned to *d*_1_ and *ϵ*_1_, then a registration transformation (rotation **Ω** and translation **τ**) between the coordinate systems of robot and laser tracker can be determined.

There are many registration techniques, one of the commonly used was developed in [30] and is based on 3D data. For calibration Method 1 described in Section 4, where only 3D data acquired by sensor are available, there is only one possible registration transformation (**Ω**, *τ*). When 6D data are available, the registration transformation can be calculated in two ways. In the first (which we name Registration (1)) **Ω**_1_ is calculated using only the 3D positional parts of full poses, as in [30]. In the second (named hereafter as Registration (2)), the rotation matrix **Ω**2 is calculated as the mean rotation $\bar{\Omega }$ calculated properly [31] from the individual matrices $G_{k}R_{k}^{-1}$ in (13). Once **Ω**_1,2_ are known, the translation vectors *τ*_1,2_ can be determined as

(19) $$\tau_{i}={\bar{g}}_{s}-\Omega_{i}\;{\bar{t}}_{r}\;,\;i=1,2$$

where ${\bar{t}}_{r}$ and ${\bar{g}}_{s}$ are the centroids of the collected 3D positions in the robot and the external sensor frame, respectively.

## 7. Simulation

All calculations were performed in Matlab. Built-in nonlinear least-square (NLS) optimizer *lsqnonlin* with default input parameters was used to minimize the error function *Err_pos_* in (10), *Err_rot_* in (17) and *Err_full_* in (18). As a starting point for all optimizations, default DH parameters were used.

To test the proposed calibration method, a kinematic model of a 7 degrees-of-freedom (DoF) industrial robot arm KUKA LWR 4+ was used. The robot’s default DH parameters (***ϵ***_0_, ***α***_0_, ***r***_0_, ***d***_0_) are provided in Table 1 (all angular parameters are in degrees and all linear in millimeters). Ground truth (GT) parameters used in simulations were defined as a sum of the defaults and deviations, for example ***ϵ**_GT_* = ***ϵ***_0_ + Δ***ϵ***. Deviations from the default DH parameters are provided in Table 2. Two sets of arbitrarily chosen deviations were used in simulations: small deviations (Δ***α***_1_, Δ***r***_1_, Δ***d***_1_) and large deviations (Δ**α**_2_, Δ***r***_2_, Δ***d***_2_). GT parameters were used to generate noisy sensor data ***G**_k_* from (7) and ***g**_k_* from (8)

(20) $$G_{k}=N_{k}\;\Omega \;R_{k}(\theta_{k},\alpha_{GT},\epsilon_{GT})$$

and

(21) $$g_{k}=\Omega t_{k}(\theta_{k},\alpha_{GT},\epsilon_{GT},r_{GT},d_{GT})+\tau +\zeta_{k}$$

where (**Ω**, *τ*) is arbitrarily selected transformation between robot and sensor frame, *ζ_k_* is 3D positional Gaussian noise with standard deviation *σ_p_*, and ***ξ**_k_* is 3D angular Gaussian noise with standard deviation *σ_a_*, which was used to generate small random rotations.


(22) $$N_{k}=R_{ZYX}(\xi_{k}).$$

**Figure 1.**
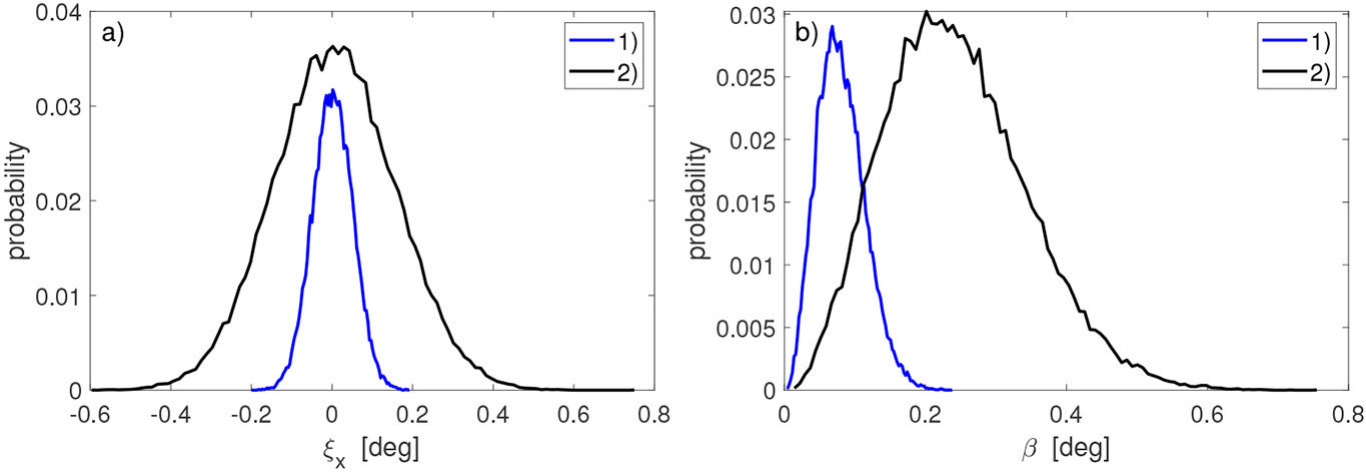
Characteristics of simulated small random rotations $N_{k}$ in (22): (**a**) histograms of *x* component of angle vectors ***ξ**_k_*; (**b**) histograms of angle of rotation *β* of rotation matrix $N_{k}$. Blue lines correspond to weak noise with *σ_a_* = 0.05° and black lines correspond to strong noise with *σ_a_* = 0.15°.

In Figure 1a, examples of histograms for *x* component of vectors *ξ_k_* are shown (histograms for *y* and *z* components look similar). In Figure 1b, histograms of corresponding angles of rotation *β* of small random rotations $N_{k}$ are plotted. Note that histograms of *ξ_k_* are well approximated by a Gaussian distribution while non-symmetric histograms of *β* are well approximated by a Fisher–Bingham–Kent (FBK) distribution [32]. Similar histograms of angles were observed for experimental data acquired with a marker-based pose measuring system, see Figures 1 and 3 in [33].

Tool transformation ***F***_*T*_ needed in (7) and (8) was arbitrarily chosen with the caveat that the TCP center is not located on the last axis of rotation so that 3D data acquired for CPA procedure are located on a circle.

For each *n*-th join, *K_α_* = 40 vectors of encoder angles *θ_n,k_* were created such that their components were all zero except *θ_n,k_*

(23) $$\theta_{n,k}=\theta_{min,n}+k\delta_{\theta },\;\;k=1,\ldots ,K_{\alpha }$$

where *θ_min,n_* and *δ_θ_* were such that all *θ_n,k_* were within a valid range of *n*-th encoder angles. These angles were then inserted in (21) to generate 3D sensor data from which the twist angles $\tilde{\alpha }$ were estimated as described in Section 3. In order to estimate the remaining DH parameters ($\tilde{\epsilon }$, $\tilde{r}$, $\tilde{d}$) and calculate registration transformation (**Ω**, **τ**), another set of *K* = 100 joint angle vectors ***θ**_k_* was selected in such a way that corresponding poses ***F**_TCP_* (***θ**_k_*) in (1) were randomly scattered in the workspace that is accessible to the robot arm. In computer simulations, this is the only restriction for selection of tool poses, but additional limitations may arise in lab experiments due to a use of a line-of-sight sensor for pose acquisition.

In addition, a separate batch of *J* = 50 joint angles *θ_j_* was selected for evaluation of calibration and registration procedures. These test poses were used neither in calibration nor registration. To test the performance of all three procedures, the robot kinematic model ***F**_TCP_* (***θ**_j_*) in (1) was used with the parameters ($\tilde{\epsilon }$, $\tilde{\alpha }$, $\tilde{r}$, $\tilde{d}$) estimated by Method 1, 2 and 3. For Method 1, the registration transformation (**Ω**, **τ**) was calculated using 3D data. For Method 2 and 3, both registrations (**Ω**_1_, **τ**_1_) and (**Ω**_2_, **τ**_2_) were calculated, as described in Section 6. For each tested arm configuration *θ_j_* and selected *m*-th noise levels (*σ_a_*, *σ_p_*)_m_, the corresponding rotation ***G**_j_* and position ***g**_j_* were calculated in (20) and (21) to simulate noisy 6D measurements acquired by sensor. Then, the mean of *J* angles ⟨*η_j_*⟩ of rotations $Q_{j}$ and the mean of *J* relative distances ⟨*q_j_*⟩ were calculated, where

(24) $$Q_{j}(\eta_{j})=G_{j}^{-1}\Omega R_{j},\;q_{j}=\|\Omega t_{j}+\tau -g_{j}\|\;,$$

and the transformation (**Ω**, **τ**) was appropriate for each of the three calibration procedures and (for Method 2 and 3) the appropriate Registration 1 or 2. Both calculated means ⟨*η_j_*⟩ and ⟨*q_j_*⟩ were used as metrics to gauge a performance of tested procedures.

These steps were repeated for each of the selected noise level *m* = 1, …, *M_n_* and both sets of GT parameters corresponding to two deviation vectors: small (Δ***α***_1_, Δ***r***_1_, Δ***d***_1_) and large (Δ***α***_2_, Δ***r***_2_, Δ***d***_2_), as shown in Table 2. *M_n_* = 16 noise levels were equally spaced between zero and 0.15 (degrees for *σ_a_* and millimeters for *σ_p_*). In order to estimate a variability of the calculated metrics, all the above calculations were repeated for *N_rep_* = 25 different realizations of noise (different sequences of pseudo-numbers). Thus, for each *i*-th instance of noise and each *m*-th pair of noise levels (*σ_a_*, *σ_p_*)_*m′*_ the end-effector errors were calculated: *v_m,i_* = ⟨*q_j_*⟩ for positional error and *ρ_m,i_* = ⟨η_j_⟩ for angular error. As the final results, the averages and standard deviations from all repeats *N_rep_* were stored for each *m*-th noise level:

(25) $${\bar{\rho }}_{m}=\frac{1}{N_{rep}}\sum_{i=1}^{Nrep}\rho_{m,i},\;\delta \rho_{m}^{2}=\frac{1}{N_{rep}}\sum_{i=1}^{Nrep}(\rho_{m,i}-{\bar{\rho }}_{m}{)}^{2}$$

and similarly for positional errors ${\bar{v}}_{m}$ and $\delta v_{m}^{2}$.

To test a performance of the three error functions *Err_pos_*, *Err_rot_* and *Err_full_* used in calibration, for a few randomly selected noise repeats and strengths, minimization was restarted from 300 randomly scattered initial points (i.e., starting DH parameters) and the final optimized parameters were analyzed. In addition, for Method 3, minimization of *Err_full_* was repeated for a few scaling factors *w* in (18).

In all simulations performed in this study, the distal variant of DH parameters was used [34]. Alternatively, the proximal variant could be used, which would affect derived from it homogeneous matrix ***F**_TCP_*. However, not every kinematic model is suitable for describing any robot: a well-known example is a robot with two consecutive joint axes that are parallel to each other. In such a case, the DH model is not continuous and must be replaced by another model, e.g., POE [20], and parameters specific for a given model must be determined. Whichever kinematic model is selected, it is important to consistently use it in a calibration process along with other basic definitions (like use of a right-hand or left-hand coordinate system). With all procedural steps clearly defined and consistently followed, there is no ambiguity in the calibration process.

## 8. Results

Fitted DH parameters revealed different amounts of variations for different simulated conditions. The twist angles $\tilde{\alpha }$ estimated from 3D data generated for the CPA procedure showed moderate variations. The largest absolute deviation *δα_max_* from the GT value over all *N* joints and all simulated conditions (*M_n_* noise levels, *N_rep_* repeats and both deviations Δ***α***_1,2_ from the default values ***α***_0_) was 0.3°. Zero offsets $\tilde{\epsilon }$ revealed larger deviations: the largest absolute deviation *δϵ_max_* = 1.18°. The largest link length deviation was *δd_max_* = 4.7 mm and the largest link offset deviation was *δr_max_* = 4.2 mm. Such large differences between the fitted and the GT parameters were observed mostly for large noise levels *σ_p_* and *σ_a_*.

**Figure 2.**
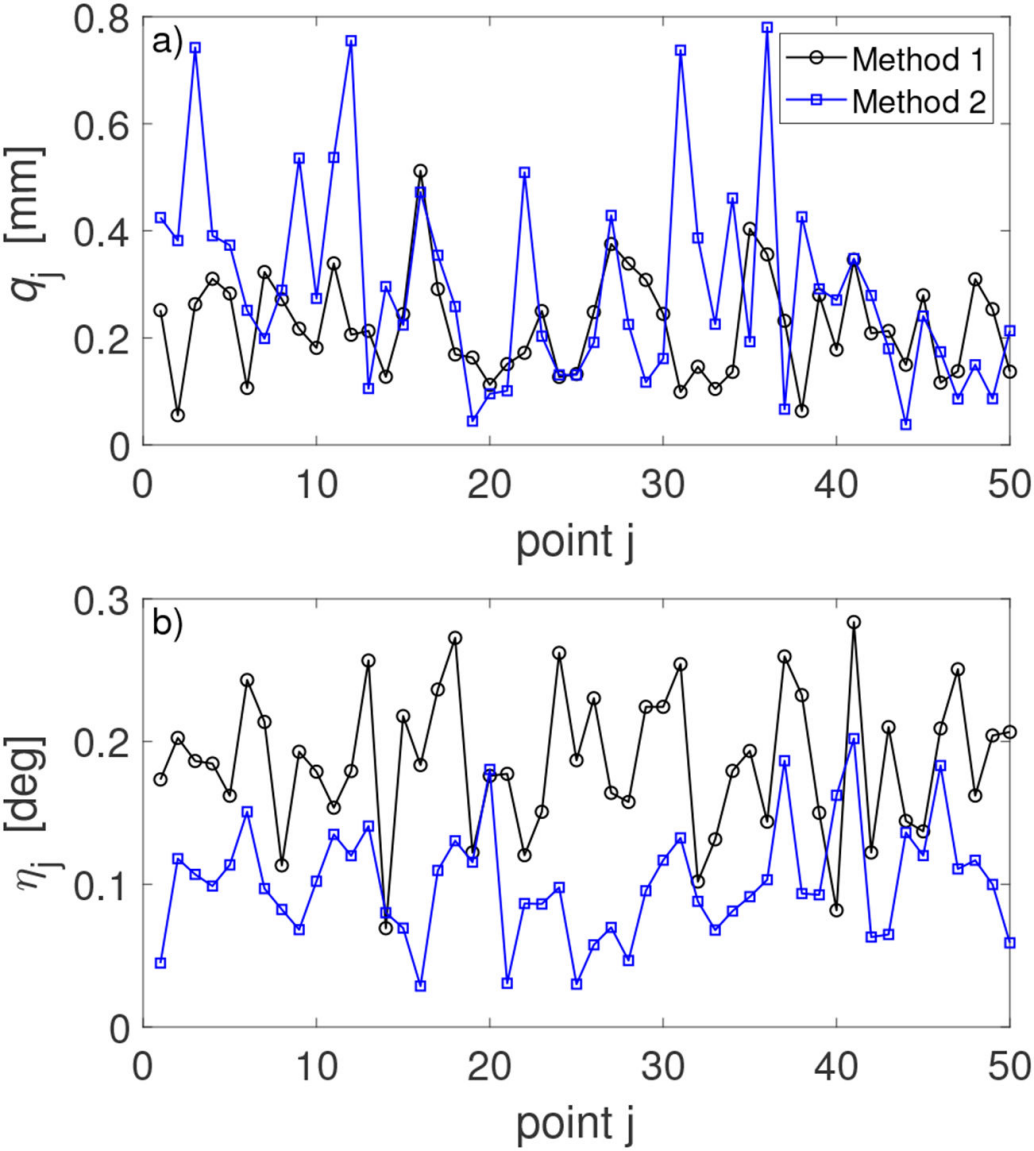
Robot end-effector errors calculated at *J* = 50 test poses for fixed sensor noise *σ_p_* = 0.055 mm, *σ_a_* = 0.055° and one, arbitrary selected noise realization: (**a**) positional errors *q_j_*; (**b**) orientation errors *η_j_*. Robot was calibrated with Method 1 (black lines) and Method 2 (blue lines).

**Figure 3.**
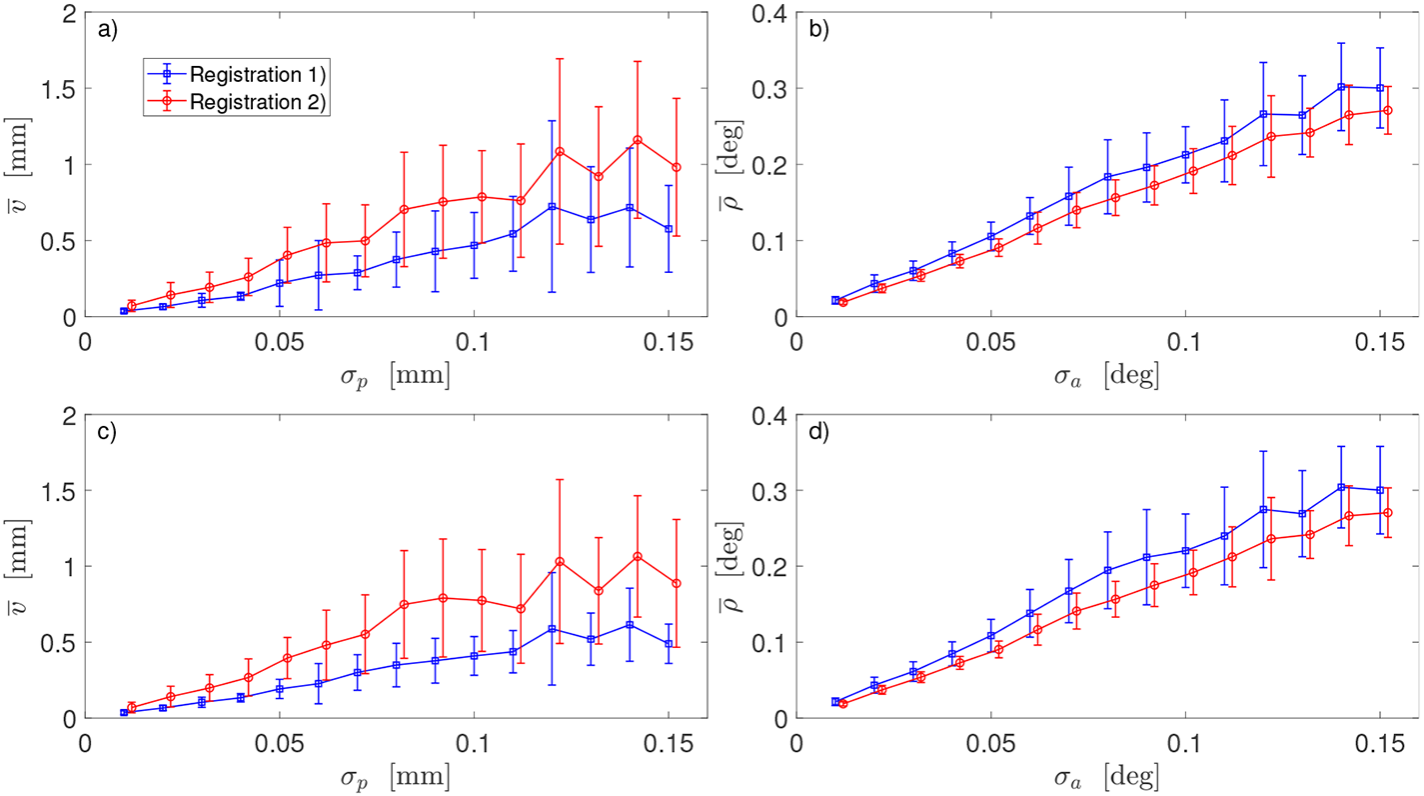
Comparison of two registration procedures for robot calibrated with Method 2 and data generated using: (**a**,**b**)—small deviations from the default DH parameters (Δ***α***_1_, Δ***γ***_1_, Δ***d***_1_); (**c**,**d**)—large deviations (Δ***α***_2_, Δ***r***_2_, Δ***d***_2_). Dependence of the mean positional error $\bar{v}$ of robot end-effector on positional noise *σ_p_* in sensor 6D data in (**a**,**d**); dependence of the mean orientation error $\bar{\rho }$ of robot end-effector on angular noise *σ_a_* in sensor 6D data in (**b**,**d**).

**Figure 4.**
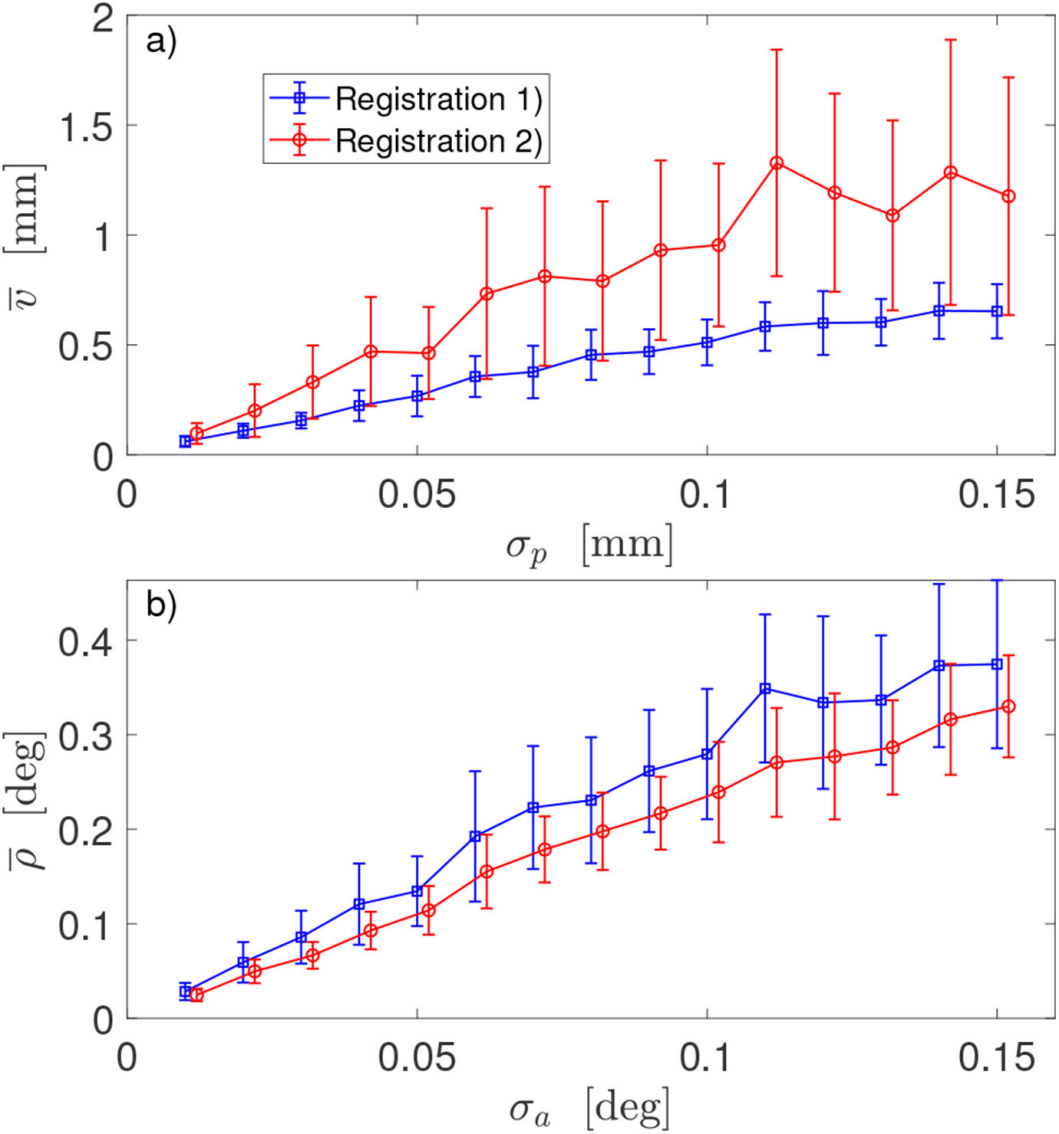
Comparison of two registration procedures for robot calibrated with Method 3 and data generated using large deviations from the default DH parameters: (**a**) dependence of the mean positional error $\bar{v}$ of robot end-effector on positional noise *σ_p_* in sensor 6D data; (**b**) dependence of the mean orientation error $\bar{\rho }$ of robot end-effector on angular noise *σ_a_* in sensor 6D data.

**Figure 5.**
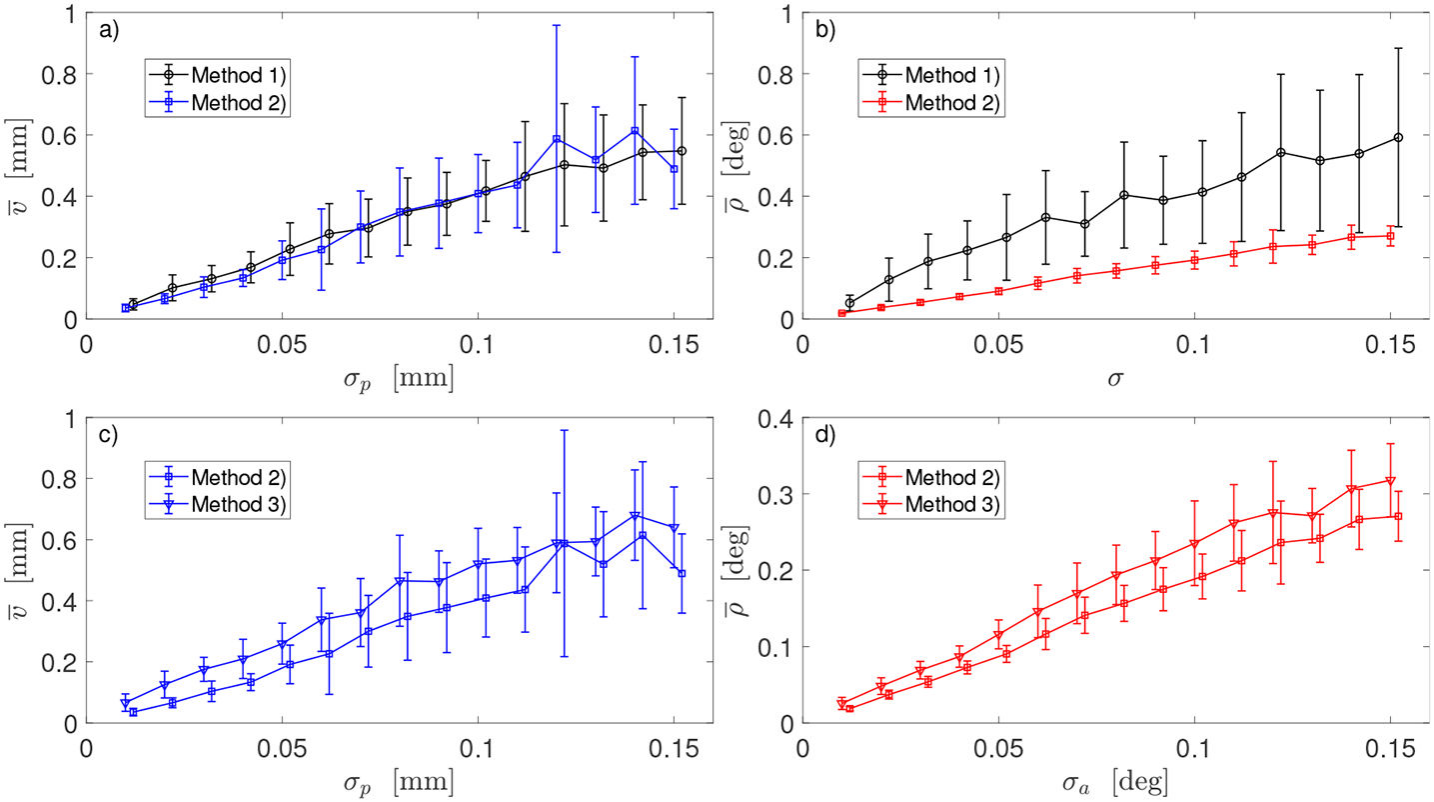
Comparison of three calibration methods: (**a**) dependence of the mean positional error $\bar{v}$ of robot end-effector on positional noise *σ_p_*—Registration 1 was used in Method 2 (blue line); (**b**) dependence of the mean orientation error $\bar{\rho }$ of robot end-effector on noise (*σ* = *σ_p_* for Method 1 and *σ* = *σ_a_* for Method 2)—Registration 2 was used in Method 2 (red line); (**c**) dependence of error $\bar{v}$ on positional noise *σ_p_*—Registration 1 was used in both methods; (**d**) dependence of error $\bar{\rho }$ on noise *σ_a_*—Registration 2 was used in both methods.

**Figure 6.**
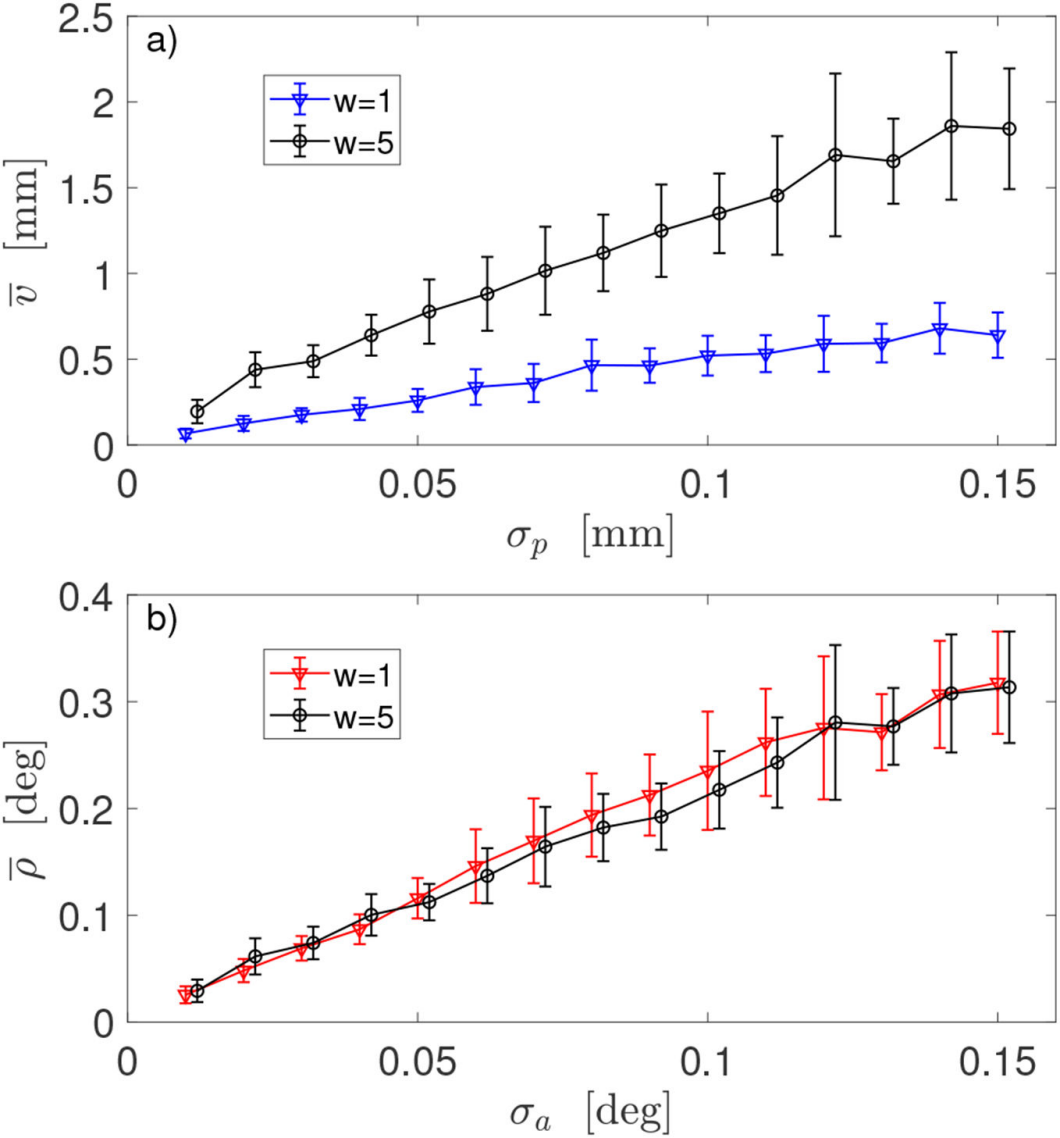
Comparison of robot calibrations using two different scaling factors *w* in error function *Err_full_* in Method 3: (**a**) dependence of the mean positional error $\bar{v}$ of robot end-effector on positional noise *σ_p_* in sensor 6D data—Registration 1 was used; (**b**) dependence of the mean orientation error $\bar{\rho }$ of robot end-effector on angular noise *σ_a_* in sensor 6D data—Registration 2 was used. Data generated using large deviations from the default DH parameters.

Figure 2 shows an example of robot end-effector errors at *J* = 50 test poses. Position errors *q_j_* and orientation errors *η_j_* were calculated in (24) for robot DH parameters calibrated with Method 1 and Method 2. Presented errors were calculated for simulated sensor poses perturbed by *i* = 14 noise realization (selected arbitrary from *N_rep_* repeats) and *m* = 7 noise levels (*σ_a_*, *σ_p_*)_*m*_. These (*q_j_*, *η_j_*)*_m,i_* errors were then used to calculate (*v_m,i_*, *ρ_m,i_*) and then, mean errors ${\bar{v}}_{m}$ and ${\bar{\rho }}_{m}$ in (25) and the corresponding standard deviations *δv_m_* and *δρ_m_* for each *m*-th noise level. These means and standard deviations were then used to create the plots in the remaining Figures 3-6.

Figure 3 shows the outcomes of two registration transformations (**Ω**_1_, **τ**_1_) and (**Ω**_2_, **τ**_2_) described in Section 6. In both cases, robot was calibrated with Method 2. GT parameters used in simulation of 6D data, i.e., end-effector poses and noisy poses as measured by sensor, were obtained by modifying the default DH parameters with deviations shown in Table 2. For both registrations, mean errors were calculated at the same values of sensor noise (*σ_p_* in Figure 3a,c and *σ_a_* in Figure 3b,d). In each subplot, two graphs are slightly shifted horizontally only for better visualization. Error bars *δv_m_* in Figure 3a,c and *δρ_m_* in Figure 3b,d are the corresponding standard deviations calculated in (25) from *N_rep_* repeated simulations of noisy sensor data.

Figure 4 shows the outcomes of two registration procedures applied after robot was calibrated using Method 3 and the error function *Err_full_* defined in (18) with the scaling factor *w* = 1. Presented results were obtained for 6D data generated with GT values of DH parameters deviating from their default values by (Δ***α***_2_, Δ***r***_2_, Δ***d***_2_) shown in Table 2.

Figure 5 shows the outcomes of three calibration procedures: Method 1 based on 3D sensor data, and Method 2 and 3 based on 6D sensor data (in Figure 5b,d noise *σ* = *σ_p_* in mm for Method 1 and *σ* = *σ_a_* in degrees for Method 2 and 3). Two different registration procedures were used in robot calibration with Method 2 and 3: for positional error $\bar{v}$, Registration 1 was used (blue line in Figure 5a,c, the same as in Figure 3c for Method 2 and the blue line with triangle markers in Figure 5c, the same as blue line in Figure 4a for Method 3). For angular error $\bar{\rho }$, Registration 2 was used (red line in Figure 5b,d, the same as in Figure 3d for Method 2 and the red line with triangle markers in Figure 5d, the same as red line in Figure 4b). Error bars *δv_m_* in Figure 5a,c and *δρ_m_* in Figure 5b,d are the corresponding standard deviations calculated in (25) from *N_rep_* repeated simulations of noisy sensor data. On each subplot, the two graphs are slightly shifted horizontally for a visualisation effect. Robot GT parameters used in simulation of 6D data were obtained by modifying the default DH parameters with large deviations (Δ***α***_2_, Δ***r***_2_, Δ***d***_2_) shown in Table 2. Similar results for $\bar{v}$ and $\bar{\rho }$ were obtained when small deviations (Δ***α***_1_, Δ***r***_1_, Δ***d***_1_) were used in simulations.

Figure 6 shows outcome of robot calibration for Method 3 with two different values of the scaling factor *w* in *Err_full_* in (18). Results for Method 3 presented in Figures 4 and 5c,d were obtained for *w* = 1.

For each of the selected cases where the minimization of the error function was repeated from 300 different starting points, all initial DH parameters led to the same solution. Fitted DH parameters depended on noise strengths, choice of error function and GT values of DH parameters.

## 9. Discussion

In this study, an open-chain robotic manipulator with *N* revolute joints was calibrated using three different methods and two different sets of data: 3D positions only, and full 6D poses. All three methods share the same strategy for determining link twists $\tilde{\alpha }$. Then, in Method 1, the error function *Err_pos_* in (10) was minimized, and the remaining DH parameters ($\tilde{\epsilon }$, $\tilde{r}$, $\tilde{d}$) were found by using 3D data only. In Method 2, a search for the zero offsets $\tilde{\epsilon }$ was performed separately by minimizing *Err_rot_* in (17), which depends only on the orientation part of full 6D data. Once the zero offsets were known, the remaining DH parameters ($\tilde{r}$, $\tilde{d}$) were found by minimizing *Err_pos_* (***r, d***) in (10) using only the positional part of 6D data. Such an approach reduces the dimensionality of the search space when compared with minimization of *Err_pos_* in Method 1. In addition, by using angles *ψ_k_* of relative rotations ***D**_k_* in error function *Err_rot_* in (17) and relative distances *L_k_* between pairs of 3D points in error function *Err_pos_* in (10), the proposed strategy decouples robot calibration from registration of the robot frame to the world frame. Different calibration strategies yielded different sets of fitted DH parameters which, in turn, led to different end-effector errors. This is expected, as the optimizer which uses different error functions and different sensor data usually converges to different solutions for the same kinematic model. It should be noted that both Methods 1 and 2 are equally valid and it is a matter of practicality which one is more useful.

In Method 2, two different approaches to registration were used. Rotation **Ω**_1_ from the first approach minimizes distances between the sensor’s 3D positions and robot’s TCP points for *K* robot arm configurations [30]. Rotation **Ω**_2_ is calculated as the mean rotation $\bar{\Omega }$ of *K* relative rotations $G_{k}R_{k}^{-1}$ and, thus, minimizes angular distances between orientations of TCP frame and orientations provided by sensor. Therefore, one may expect that **Ω**_2_ is better than **Ω**_1_ in aligning robot orientations with sensor orientations. Indeed, end-effector angular errors $\bar{\rho }$ shown in Figure 3b,d are smaller for **Ω**_2_ in Registration 2 (red line) than for **Ω**_1_ in Registration 1 (blue line).

When it comes to the positional errors $\bar{v}$, the situation is exactly opposite. Both translation vectors **τ**_1_ and **τ**_2_ are calculated in (19). Since **Ω**_2_ does not depend on positional data, the transformation (**Ω**_2_, **τ**_2_,) does not minimize (in the least-square sense) the distances between sensor 3D positions and robot TCP points for *K* robot arm configurations. Transformation (**Ω**_1_, *τ*_1_,) does minimize the distances, and therefore is expected to better align the sensor 3D positions with the robot TCP. Indeed, end-effector position errors $\bar{v}$ shown in Figure 3a,c are smaller for **Ω**_1_ in Registration 1 (blue line) than for **Ω**_2_ in Registration 2 (red line).

Analysis of the plots in Figure 3 suggests the optimal strategy: instead of choosing either the first (**Ω**_1_, *τ*_1_,) or the second (**Ω**_2_, **τ**_2_,) registration, take the best part from both. Use **Ω**_2_ to transform orientations ***R**_k_* of robot end-effector and use (**Ω**_1_, **τ**_1_,) to transform robot TCP ***t**_k_*. Outcome of such strategy is displayed in Figure 5a,b: note blue line for positional errors $\bar{v}$ and red line for angular error $\bar{\rho }$ indicating a use of different registrations in Method 2.

Another advantage of using **Ω**_2_ to transform the TCP orientations rather than **Ω**_1_ is that there is a much smaller dispersion of orientation errors $\bar{\rho }$ for different noise realizations. The error bars in Figure 5b are much smaller for Method 2 (which leverages **Ω**_2_) than for Method 1. This implies that orientations from the world coordinate system can be fed into an inverse kinematic solver more consistently and accurately.

It may appear counter intuitive that mean position errors $\bar{v}(\sigma_{p})$ and mean orientation errors $\bar{\rho }(\sigma_{a})$ calculated for the same m-th pair of noise strengths (*σ_p_*, *σ_a_*)_*m*_ but different GT values of DH parameters are almost the same, as Figure 3 shows. However, it should not be a surprise since we used NLS optimizer with exact error function. Scale of deviation from the default DH parameters may become an issue when the calibration is performed using approximated, linearlized errors and the Jacobian is calculated at the default DH values.

Results of robot calibration obtained with Method 3 clearly reveal the consequences of scaling problem when simultaneous minimization of both position and orientation errors in one optimization is attempted, as demonstrated in Figure 6. While the mean orientation errors $\bar{\rho }$ are almost equal for two selected values of *w*, the corresponding position errors $\bar{v}$ differ substantially. This method, similarly as Method 2, uses 6D data and, therefore, two registration procedures are available. In Method 3, similarly to Method 2, smaller position errors are obtained when Registration 1 is applied to the position data and smaller orientations errors are observed when Registration 2 is applied to the orientation data, as results in Figure 4 clearly indicate. Even as both Method 2 and 3 share a possibility of using different registrations for position and rotation components of a full pose, a direct comparison between the two methods clearly points to Method 2 as a better procedure, as demonstrated by the results shown in Figure 5c,d. Thus, a use of Method 3 is discouraged.

The calibration strategy outlined in this paper was tested on a kinematic model of a serial open chain robot with revolute joints only. A question can be asked if the strategy can be applied to a more complex kinematic model when a serial chain has both revolute and prismatic joints. Acquisition of full 6D poses enables calculation of two registrations defined in (19): one of them minimizes a position error and the other minimizes an orientation error. Therefore, as long as full 6D poses are acquired, the outlined calibration strategy could in principle be used for robots with a mixture of revolute and prismatic joints. However, a presence of prismatic joints complicates the error function *Err_pos_* in (10) by increasing a number of search variables and it requires further study to verify whether the strategy is beneficial also for robots with revolute and prismatic joints.

The simulation results presented in this paper raise an important, practical question about the characteristics of 6D pose measuring sensors which are used for robot calibration. Commercially available sensors allow quick acquisition of many repeated measurements, which enables the noise in recorded data to be substantially reduced by calculating mean poses. The mean position error of robot end-effector $\bar{v}$ calculated by Method 2 is increasing with sensor position noise *σ_p_*, as Figure 5a shows. If the three sigma rule is followed and approximate relation $\bar{v}\approx 4\sigma_{p}$ holds, then the upper bound ${\hat{\sigma }}_{p}$ for sensor position noise should satisfy $12{\hat{\sigma }}_{p}\;<\;tol_{p}$, where *tol_p_* is the acceptable robot position tolerance. For orientation data, due to the strong non-symmetric FBK-like distribution of angles *β* (which accounts for deviation of noisy, instantaneous rotations from the mean rotation), the three sigma rule can be replaced by calculating quantile ${\hat{\beta }}_{997}$ of angles *β* at 0.997 level. Assuming the mean orientation error of robot end-effector $\bar{\rho }$ is four times larger than sensor’s orientation noise (as shown in Figure 5b for Method 2), the upper bound for sensor orientation noise should satisfy $4{\hat{\beta }}_{997}<tol_{a}$ where *tol_a_* is the acceptable robot orientation tolerance. For different robot, the dependence of end-effector error on sensor noise may be different from that shown in Figure 5a,b. Then, the estimates for upper bounds of position noise ${\hat{\sigma }}_{p}$ and orientation noise ${\hat{\beta }}_{997}$ need to be updated.

The proposed calibration strategy reduces both the position and orientation errors of the robot end-effector. Recommended procedure for serial robot calibration consists of: (1) acquiring the full 6D poses; (2) getting link twists in CPA-like procedure; (3) getting encoder zero offsets using orientation data only; (4) getting link lengths and offsets using position data only. Then, use two separate registrations to transform position and orientation component of a pose from a world to the robot frame. In summary, the dilemma of having only the position or the orientation error of the robot’s end-effector minimized can be avoided and a pose with both optimized components can be fed into inverse kinematic solver.

## Figures and Tables

**Table 1.** Default DH parameters.

	1	2	3	4	5	6	7
***ϵ***_0_	180	90	0	0	0	0	0
***α***_0_	−90	−90	−90	90	90	−90	0
***r***_0_	0	0	0	0	0	0	0
***d***_0_	310.5	0	400	0	390	0	78

**Table 2.** Deviations of DH parameters.

	1	2	3	4	5	6	7
Δ***ϵ***	0	−1.4	0.68	0.24	0.54	1.37	0.85
Δ***α***_1_	0.15	−0.1	0.07	−0.04	0.02	−0.06	0
Δ***α***_2_	3.35	−4.1	2.7	−3.4	4.2	−3.6	0
Δ***r***_1_	0.25	0.4	0.09	0.3	0.28	0.17	0.06
Δ***r***_2_	0.8	1.3	0.65	1.4	0.86	0.38	0.55
Δ***d***_1_	0	0.06	−0.14	0.12	0.27	0.08	0.3
Δ***d***_2_	0	0.27	−1.45	0.4	1.26	0.3	0.35
